# The probability of receiving a kidney transplantation in end-stage kidney disease patients who are treated with haemodiafiltration or haemodialysis: a pooled individual participant data from four randomised controlled trials

**DOI:** 10.1186/s12882-021-02265-6

**Published:** 2021-02-25

**Authors:** Robin W. M. Vernooij, Way Law, Sanne A. E. Peters, Bernard Canaud, Andrew Davenport, Muriel P. C. Grooteman, Fatih Kircelli, Francesco Locatelli, Francisco Maduell, Marion Morena, Menso J. Nubé, Ercan Ok, Ferran Torres, Mark Woodward, Peter J. Blankestijn, Michiel L. Bots, on behalf of the HDF Pooling project investigators

**Affiliations:** 1 Department of Nephrology and Hypertension, University Medical Center Utrecht, Utrecht, the Netherlands; 2 Julius Center for Health Sciences and Primary Care, University Medical Center Utrecht, Utrecht University, Utrecht, the Netherlands; 3 Wai-ping Law, Renal unit, Department of medicine, Queen Elizabeth Hospital, Hong Kong, PR China; 4 The George Institute for Global Health, School of Public Health, Imperial College, London, UK; 5 The George Institute for Global Health, University of New South Wales, Sydney, Australia; 6 Global Medical Office, Fresenius Medical Care Deutschland, Bad Homburg, Germany; 7 Montpellier University, School of Medicine, Montpellier, France; 8 University College London, Centre for Nephrology, Royal Free Hospital, London, UK; 9 Department of Nephrology and Amsterdam Cardiovascular Sciences (ACS), Amsterdam University Medical Centers, VU University, Amsterdam, The Netherlands; 10 Division of Nephrology, Ege University School of Medicine, Izmir, Turkey; 11 Department of Nephrology, Alessandro Manzoni Hospital, past director, Lecco, Italy; 12 Nephrology Department, Hospital Clinic, Barcelona, Spain; 13 PhyMedExp, University of Montpellier, INSERM, CNRS, Biochemistry/Hormonology department, University Hospital Center of Montpellier, Montpellier, France; 14 Biostatistics Unit, School of Medicine, Universitat Autònoma de Barcelona, Barcelona, Spain; 15 Medical Statistics core facility, IDIBAPS, Hospital Clinic, Barcelona, Spain; 16 Department of Epidemiology, Johns Hopkins University, Baltimore, MD USA

## Abstract

**Background:**

Due to a critical shortage of available kidney grafts, most patients with Stage 5 Chronic Kidney Disease (CKD5) require bridging dialysis support. It remains unclear whether treatment by different dialysis modalities changes the selection and/or preparation of a potential transplant candidate. Therefore, we assessed whether the likelihood of receiving kidney transplant (both living or deceased kidney donors) differs between haemodialysis (HD) and online haemodiafiltration (HDF) in patients with CKD5D.

**Methods:**

Individual participant data from four randomised controlled trials comparing online HDF with HD were used. Information on kidney transplant was obtained during follow-up. The likelihood of receiving a kidney transplant was compared between HD and HDF, and evaluated across different subgroups: age, sex, diabetes, history of cardiovascular disease, albumin, dialysis vintage, fistula, and level of convection volume standardized to body surface area. Hazard ratios (HRs), with corresponding 95% confidence intervals (95% CI), comparing the effect of online HDF versus HD on the likelihood of receiving a kidney transplant, were estimated using Cox proportional hazards models with a random effect for study.

**Results:**

After a median follow-up of 2.5 years (Q1 to Q3: 1.9–3.0), 331 of the 1620 (20.4%) patients with CKD5D received a kidney transplant. This concerned 22% (*n* = 179) of patients who were treated with online HDF compared with 19% (*n* = 152) of patients who were treated with HD. No differences in the likelihood of undergoing a kidney transplant were found between the two dialysis modalities in both the crude analyse (HR: 1.07, 95% CI: 0.86–1.33) and adjusted analysis for age, sex, diabetes, cardiovascular history, albumin, and creatinine (HR: 1.15, 95%-CI: 0.92–1.44). There was no evidence for a differential effect across subgroups based on patient- and disease-characteristics nor in different categories of convection volumes.

**Conclusions:**

Treatment with HD and HDF does not affect the selection and/or preparation of CKD5D patients for kidney transplant given that the likelihood of receiving a kidney transplant does not differ between the dialysis modalities. These finding persisted across a variety of subgroups differing in patient and disease characteristics and is not affected by the level of convection volume delivered during HDF treatment sessions.

## Introduction

Kidney transplantation is the treatment of choice for patients with Stage 5 Chronic Kidney Disease (CKD5). Yet, due to an aging population and an increased prevalence of CKD5 in patients with diabetes and hypertension [1], a critical shortage exists of available kidney grafts for transplantation [2, 3]. Consequently, most patients with CKD5 require bridging dialysis support (CKD5D). The role of the different dialysis modalities, i.e. haemodialysis (HD), peritoneal dialysis (PD), or haemodiafiltration (HDF), with respect to the selection and/or preparation of receiving kidney transplantation has been the topic of persistent discussion [4–6]. While most previous research regarding the selection and/or preparation of kidney transplantation candidates focussed on the comparison between PD and HD [7–9], limited evidence is available regarding the comparison of HDF and HD [10, 11]. While the convection volume in standard HD approximates 10 L/session, in online post-dilution HDF the convection volume may amount to 23 L/1.73m^2^/session. Online HDF has been associated with a lower all-cause and cardiovascular mortality risk when compared with HD, especially for the patients receiving the highest delivered convection volume [12]. Yet, so far it is unclear whether HDF influences the eligibility and preparation time for receiving a renal transplant in these patients.

The choice of dialysis modality might potentially affect the selection or preparation of potential kidney transplant candidates due to several differences between dialysis modalities. For example, several aspects of immune function and cytokine production, such as (micro) inflammation and oxidative stress [7, 13], the hemodynamic treatment tolerance [11], and risk of intradialytic hemodynamic instability, and hence, injury to vital organs, including the heart and brain, vary between dialysis modalities [7, 14]. Additionally, HD membranes might increase free radical production by activating complement factors, phagocytes, and leucocytes [14, 15]. At the same time, patients might have been selected for a specific dialysis modality due to better prognostic factors (e.g. age or comorbidities) that can also affect the likelihood of receiving a kidney transplantation. Therefore, the aim of our study was to examine, in a pooled individual patient data analysis of four randomised controlled trials, first, whether the likelihood of receiving transplantation differs between HDF and HD, and second, whether the probability of receiving a kidney transplantation differed according to patient-, disease-, or other treatment-related characteristics.

## Methods

### Study design and study populations

A pooled individual participant data (IPD) meta-analysis was undertaken using data from four large multicentre randomised controlled trials (RCTs) comparing online HDF versus HD in adult patients with CKD5 receiving chronic HD [12]. All methods carried out and reported in accordance with relevant guidelines [16, 17]. Detailed description of the study designs, including eligibility criteria and treatment procedures, are provided elsewhere [18–21]. Briefly, the CONTRAST study included 714 patients treated by HD for > 2 months in dialysis centres in the Netherlands, Canada, and Norway. A suggested target convection volume of 6 L/hour, i.e. generally 24 L/per session, was proposed [18]. The ESHOL study included 906 patients treated by HD for > 3 months in Spain, with a minimum of 18 L/session of convection volume for the patients treated with online HDF [20]. The French HDF study included 391 patients, aged ≥65 years, treated by HD for > 1 month, with no target online HDF convection volume specified [21]. The Turkish HDF study, including 782 patients without a specification of HD vintage, with a minimum target of 15 L/session convection volume for online HDF treatments [19]. In all studies patients were 1:1 randomised to either continuation of HD or online HDF, generally in a thrice weekly treatment schedule. In the CONTRAST study, patients in the control group were dialysed using low flux membranes, in the other three studies high flux membranes were used.

### Study endpoints and follow-up

The primary outcome differed across studies: from all-cause mortality [18, 20], a composite outcome of all-cause mortality and nonfatal cardiovascular events [19], to intradialytic tolerance [21]. Secondary outcomes collected were cardiovascular mortality, infection mortality, and sudden death. The outcome of interest for this study is kidney transplant, for which information was collected in all studies during follow-up.

### Statistical analysis

A similar statistical approach as the original IPD meta-analysis has been followed [12]. Hazard ratios (HRs), with corresponding 95% confidence intervals (95% CI), comparing the effect of online HDF versus HD on the suitability of kidney transplant candidates were estimated using Cox proportional hazards models with a random effect for study. Further analyses were conducted to evaluate whether this differed between subgroups on age (< 65 vs. ≥ 65 years), sex, history of diabetes, history of CVD, albumin levels (< 4 vs. ≥ 4 g/dL), dialysis vintage (< 30 vs. ≥ 30 months), and mode of vascular access (arteriovenous fistula vs other). We used multiplicative interaction terms to explore whether differences in effect were present across subgroups. Additionally, study-specific results were computed for the CONTRAST and ESHOL studies; the other trials had too few patients to be able to obtain precise estimates. The dose-response association between online HDF and kidney transplantation was examined by thirds of the actual (on-treatment) delivered, 1.73m^2^ BSA (body surface area)-standardized, convection volume. BSA was estimated using the formula from Gehan and George, as recommended by the European Best Practice Guidelines. Delivered convection volume was standardized by dividing the delivered by patient BSA [1.73 * (patient convection volume/patient BSA)]. The association between convection volume and likelihood of receiving kidney transplantation was adjusted for age, sex, baseline serum albumin, creatinine, history of diabetes and history of CVD. Complete case analyses were conducted given that the specific data for (time to) transplantation was almost complete (*n* = 1 missing). All analyses were performed using R (version 3.5.1.) and a two-sided *p* value of < 0.05 conferred statistical significance.

## Results

Of the 2793 patients included, 355 (12.7%) underwent a kidney transplantation during a median follow-up of 2.5 years (Q1-Q3: 1.9–3.0 years). In the CONTRAST and ESHOL study, respectively, 21% (*n* = 151) and 20% (*n* = 180) of the patients included received a kidney transplant during follow-up, but in the French and Turkish studies these percentages were much smaller (*n* = 7 and *n* = 17, respectively, both 2%) (Appendix 1).

Patients who received a kidney transplant during follow-up were, on average, younger and had fewer comorbidities compared with those patients who did not receive a kidney transplant, but no differences by sex were found (Table 1). Patients who received a kidney transplant had significantly less dialysis vintage and more arteriovenous fistula access compared with patients who did not receive a kidney transplant. Albumin, haemoglobin, and pre-dialysis serum creatinine were greater, while C-reactive protein was lower, for those patients who received a transplant. There were no apparent differences in baseline characteristics, comorbidities, or laboratory measurements between the HD and the HDF study treatment arm for those patients who underwent a kidney transplantation.

**Table 1:** Baseline characteristics of study participants by treatment allocation and transplantation during follow-up

	HD			HDF			
	No transplantation	Transplantation	***p***-value**	No transplantation	Transplantation	***p***-value**	***p***-value***
N	**654**	**152**		**635**	**179**		
Age (year)	67.8 (13.1)	54.3 (12.3)	< 0.01	67.4 (13.2)	53.6 (12.3)	< 0.01	0.58
Women	227 (34.7)	59 (38.8)	0.39	219 (34.5)	64 (35.8)	0.82	0.65
History of CVD	284 (43.4)	32 (21.1)	< 0.01	260 (40.9)	35 (19.6)	< 0.01	0.84
Diabetes mellitus	176 (27.5)	24 (16.1)	< 0.01	163 (26.0)	33 (18.4)	0.05	0.68
Dialysis vintage (months)*	27.0 (12.0–55.0)	25.5 (11.8–48.0)	0.23	26.0 (12.0–56.0)	24.0 (12.0–42.0)	0.16	0.69
Systolic BP (mmHg)	141.7 (24.3)	141.6 (21.5)	0.96	141.0 (23.9)	141.6 (21.4)	0.79	0.98
Diastolic BP (mmHg)	72.4 (14.8)	77.0 (11.6)	< 0.01	72.6 (13.5)	78.8 (12.9)	< 0.01	0.18
Vascular access, AVF	520 (79.5)	140 (92.1)	< 0.01	526 (82.8)	160 (89.4)	0.04	0.51
Duration of a dialysis session (min)	230.7 (22.0)	232.1 (20.6)	0.45	230.7 (20.8)	234.2 (21.3)	0.05	0.38
Blood flow (ml/min)*	350 (300–400)	350 (300–400)	0.82	350 (320–400)	350 (300–400)	0.35	0.27
Dialysis single pool Kt/V	1.52 (0.30)	1.53 (0.31)	0.92	1.6 (0.3)	1.5 (0.3)	0.30	0.83
Hemoglobin (g/dL)	11.9 (1.4)	11.9 (1.2)	0.41	11.9 (1.4)	12.2 (1.3)	< 0.01	0.07
Phosphorus (mg/dL)	4.8 (1.5)	5.0 (1.4)	0.14	4.8 (1.5)	5.2 (1.5)	< 0.01	0.08
B-2 microglobulin (mg/L)*	25.9 (19.8–36.2)	28.5 (20.9–38.1)	0.43	25.8 (18.8–34.9)	26.7 (18.7–34.3)	0.88	0.19
BMI after dialysis (kg/m^2^)	25.4 (4.6)	24.6 (4.0)	0.06	25.1 (5.0)	25.1 (4.0)	0.97	0.25
Albumin (g/dL)	4.0 (0.4)	4.2 (0.4)	< 0.01	4.1 (0.4)	4.2 (0.4)	< 0.01	0.61
C-reactive protein (mg/L)*	5.7 (3.4–12.8)	4.9 (1.6–8.4)	< 0.01	6.2 (3.1–14.1)	4.9 (1.6–9.9)	< 0.01	0.51
Predialysis creatinine (mg/dL)	8.7 (2.7)	9.7 (2.8)	< 0.01	8.4 (2.7)	9.9 (2.7)	< 0.01	0.56
Cholesterol (mmol/L)	3.7 (1.0)	3.7 (1.2)	0.90	3.7 (0.9)	3.6 (0.9)	0.48	0.59
Body Surface Area (m^2^)	1.8 (0.2)	1.8 (0.2)	0.62	1.8 (0.2)	1.8 (0.2)	0.42	0.96
Convection volume (L)*	–	–	–	22.8 (19.8–25.4)	22.9 (20.0–25.9)	0.40	–

Values are n (%) for categorical variables, and mean (standard deviation) or *median (Q1-Q3) for continuous variables

** *p*-values indicates differences between the patients who did not receive transplantation versus those who did in, respectively, the HD and HD arm

*** *p*-values indicates differences between the patients who received transplantation in the HD arm versus the patients who received transplantation in the HDF arm

*CVD* cardiovascular disease, *BP* blood pressure, *AVF* arteriovenous fistula, *BMI* body mass index

The patients who received online HDF did not have a different likelihood of receiving a kidney transplantation compared with the patients receiving HD (HR: 1.07, 95%-CI: 0.86–1.33) (Table 2). When adjusted for age, sex, diabetes, cardiovascular history, albumin, and creatinine, this finding remained similar (HR: 1.15, 95%-CI: 0.92–1.44). This was consistent across all studies (Appendix 2). No evidence was found for a differential effect of online HDF compared to HD in subgroup of patients differing in sex, age, diabetes, CVD history, albumin, dialysis vintage, and vascular access for the likelihood of receiving kidney transplantation.

**Table 2:** Hazard ratio and 95% confidence intervals for receiving transplantation comparing HDF versus HD, overall and in predefined strata

		Transplantation	***P***-value for interaction
	**Overall**	1.07 (0.86–1.33)	**–**
**Sex**	Male	1.01 (0.77–1.33)	
	Female	1.21 (0.85–1.73)	0.42
**Age**	< 60 years	1.27 (0.96–1.68)	
	≥ 60 years	0.87 (0.62–1.23)	0.09
**Diabetes**	No	1.05 (0.83–1.34)	
	Yes	1.26 (0.74–2.13)	0.54
**History of CVD**	No	1.17 (0.92–1.50)	
	Yes	0.88 (0.55–1.43)	0.30
**Albumine (g/dL)**	< 4	1.09 (0.72–1.64)	
	≥ 4	1.06 (0.82–1.38)	0.92
**Creatinine (mg/dl), predialysis**	< 8	0.91 (0.60–1.37)	
	≥ 8	1.14 (0.87–1.47)	0.37
**Dialysis vintage**	< 30 months	1.13 (0.85–1.50)	
	≥ 30 months	1.00 (0.71–1.40)	0.58
**Fistula**	No	1.44 (0.70–2.97)	
	Yes	1.04 (0.83–1.31)	0.40

When compared to HD, the HRs by increasing delivered convection volume (< 19, 19–23, and > 23 l per 1.73m^2^ BSA per session) were 1.15 (0.82–1.61), 1.02 (0.75–1.41) and 1.06 (0.80–1.40), respectively (Table 3). The crude analyses and analyses adjusted for age, albumin, creatinine, history of CVD, and history of diabetes, give similar results.

**Table 3:** Hazard ratio and 95% confidence intervals for transplantation by delivered BSA-standardized convection volume in litres per 1.73m^2^ per treatment with standard haemodialysis as a reference

	HD	HDF convection volume		
	–	< 19	19–23	> 23
Total patients	806	211	230	359
Number of renal transplantations	152 (18.9%)	49 (23.2%)	52 (22.6%)	77 (21.4%)
Crude	ref.	1.15 (0.82–1.61)	1.02 (0.75–1.41)	1.06 (0.80–1.40)
Adjusted^a^	ref.	1.35 (0.95–1.90)	1.15 (0.82–1.60)	1.06 (0.79–1.41)

^a^Adjusted for age, sex, albumin, creatinine, history of cardiovascular diseases, and diabetes. Values are hazard ratios and 95% confidence intervals

## Discussion

In this pooled IPD analysis including four RCTs, we identified no differences between pre-transplantation treatment with HD compared with pre-transplantation HDF in the likelihood of receiving kidney transplant in patients with CKD5D. There was no evidence to suggest that this finding differed across subgroups of patients, nor did the delivered convection volume per sessions influence this finding.

Due to an international shortage of available kidney grafts, many patients with CKD5D in clinical practice will have to undergo bridging dialysis support. Given that the underlying physiology of the different forms of dialysis varies, i.e. where HD mainly relies on diffusion, HDF consists of both diffusion and convection and is more efficient in removing middle molecules [22], this might have resulted in differences which could have affected patient suitability for kidney transplantation. Similarly, the survival benefit of HDF, i.e. a lower all-cause and cardiovascular mortality [12, 23–25], might also potentially contribute to differences selection and/or preparation of kidney transplant candidates. Patients treated with HDF have a better preservation of left ventricle function [24, 26], and less intradialytic hypotensive episodes [11, 20, 27], which might preclude or defer patients from receiving a kidney transplantation. Consequently, taking these reasons into account, one could hypothesise that pre-transplantation treatment with HDF might make EKSD patients more suitable for a kidney transplantation. However, in this study, due to randomisation, we were able to exclude measured and unmeasured confounding by indication on better prognostic factors (e.g. age and comorbidities) that affect the likelihood of receiving a kidney transplantation. At the same time, there is no hypothesis that major determinants of transplant candidate selection, such as donor specific antibodies or human leukocyte antigens (HLA) matching, will be affected by the specific dialysis modality. Therefore, we conclude that the decision-making of patient suitability for kidney transplant does not depend on the specific choice of dialysis modality.

In the French and the Turkish study, both part of the IPD meta-analysis, the proportion of patients receiving a kidney transplantation during follow-up was very low (2%) and therefore not included in our main analysis. This might be explained by several factors related to either local healthcare policy, cultural differences, or patient characteristics and personal intention. For example, although the rate of kidney transplant per million inhabitants is higher in Turkey than the rest of Europe, this is largely caused by a higher rate of living kidney transplants [28]. Given that the majority of kidney transplants in our study come from deceased donors and not living donors, this might explain the low transplantation rate in this study. On the other hand, the average age of the patients included in the French study was 76 years due to advanced age being a selection criterion [21], compared with the other studies (mean age: 64.1 years, 65.4 years, and 56.5 years in, respectively, CONTRAST, ESHOL, and Turkish study) [18–20], meaning that most transplant candidates were excluded. Consequently, the French and Turkish studies were underpowered to analyse the likelihood of receiving a kidney transplant. The criteria of kidney transplantation were according to the local policies (e.g. guidelines) of the countries of the included studies. Given the pragmatic nature of all trials included, this does not differ from actual clinical practice.

Most of the previous studies on the likelihood of receiving a kidney transplant has focussed on the comparison of HD versus PD, and the role of HDF in access to transplantation has rarely been investigated. One meta-analysis of Wang et al., including six studies with 2727 patients, reported a borderline significant increase (RR: 1.20, 95% CI: 1.00–1.44) in the likelihood of receiving a kidney transplant among patients treated with HDF compared to those patients treated with HD [10]. However, the limitations of this review are aggregated data, without subgroup analyses. These findings are contradicted by an observational registry study, including 28,407 patients (median follow-up: 1.95 years), that found that patients treated by HDF received fewer kidney transplants compared with HD patients [29].

We were unable to examine post-transplantation patient and graft survival due a limited follow-up after transplantation (median time of follow-up post-transplantation: 24 months), limited number of post-transplantation events, and differences in transplantation rates between countries. The different effects between HD and HDF in terms of oxidative stress, immune function, and hemodynamic treatment tolerance, on the success of kidney transplantation remain an interesting area of research. There are multiple studies that have compared post-transplantation outcomes [30–41]. Yet, these studies all compared the post-transplantation outcomes for patients who underwent pre-transplantation treatment with PD versus HD. Therefore, it remains unclear as to whether the choice of pre-dialysis treatment modality affects post-transplantation survival, and future research is required to elucidate this further.

The strengths of our study are that we analysed IPD from the largest available set of RCTs and made appropriate adjustment for measured and unmeasured confounders. Consequently, we produced the most reliable evidence on whether the likelihood of receiving a kidney transplant differed between HD and HDF for patients with CKD5D. However, our study is subject to some limitations. The four trials were not primarily designed to study the likelihood of patients receiving kidney transplants, since the primary end-point was mortality and differences between trials in terms of study design and methodology, and in- and exclusion criteria of patients remain. Therefore, we do not draw any causal conclusions in terms of renal transplantation and dialysis modalities. We explored these clinical differences in a wide range of subgroup analyses, which illustrated that our findings were robust for all explored factors, including patient characteristics and comorbidities. This might be limited by the fact that our study population consisted largely of European Caucasian patients. Finally, the average follow-up duration in the studies in our IPD dataset was approximately 2.5 years [12], while the average waiting time for kidney transplantation in Europe might be longer [42, 43], and the median waiting time in US is 3.6 years [3]. Potentially, a greater number of patients could have received a kidney transplant after the end of our data collection, however, it is unlikely that this would influence our findings given that we have not found any difference in the likelihood of undergoing kidney transplantation between the two treatment modalities in any subgroup or the level of convection volume delivered during treatment sessions.

In conclusion, online HDF, compared with conventional HD, does not affect the likelihood of receiving a kidney transplant. This finding holds across a variety of important clinical subgroups and is not affected by the level of convection volume delivered during HDF treatment sessions.

## Data Availability

Requests for access to the data of the HDF pooling project can be directed the corresponding author. Requests will be handled by the executive committee of the HDF pooling project.

## Appendix 1

**Table 4:** Baseline characteristics of the patients who received transplantation during follow-up stratified by study

	CONTRAST	ESHOL	French study	Turkish study	***p***-value
N	**151 (21.1)**	**180 (19.6)**	**7 (1.8)**	**17 (2.2)**	
Age (year)	53.4 (12.3)	54.4 (12.3)	67.7 (2.7)	44.5 (12.9)	< 0.001
Female sex	63 (41.7)	60 (33.3)	3 (42.9)	7 (41.2)	0.446
History of CVD	33 (21.9)	34 (18.9)	–	3 (33.3)	0.364
Diabetes mellitus	30 (20.3)	27 (15.0)	1 (16.7)	5 (29.4)	0.372
Dialysis vintage (months)*	26.0 (14.0–44.0)	23.0 (10.8–44.3)	32.3 (21.7–48.7)	55.8 (27.8–75.0)	0.013
Systolic BP (mmHg)	147.6 (21.5)	136.5 (20.0)	141.7 (24.6)	125.9 (11.0)	< 0.001
Diastolic BP (mmHg)	80.0 (10.7)	76.3 (13.3)	72.5 (5.8)	74.7 (7.8)	0.017
Vascular access, AVF	130 (86.1)	170 (94.4)	3 (42.9)	15 (88.2)	< 0.001
Duration of a dialysis session (min)	227.6 (22.1)	237.8 (18.9)	225.0 (36.7)	237.5 (3.6)	< 0.001
Blood flow (ml/min)*	300 (275–325)	400 (350–400)	310 (300–335)	309.2 (297.4–346.3)	< 0.001
Dialysis single pool Kt/V	1.4 (0.2)	1.7 (0.3)	1.7 (0.2)	1.4 (0.3)	< 0.001
Hemoglobin (g/dL)	11.9 (1.1)	12.2 (1.3)	11.0 (1.4)	11.8 (1.3)	0.013
Phosphorus (mg/dL)	5.2 (1.5)	5.0 (1.4)	4.5 (1.9)	4.9 (1.1)	0.291
B-2 microglobulin (mg/L)*	32.3 (24.6–40.7)	20.9 (16.3–26.9)	23.0 (21.0–27.2)	26.2 (23.9–28.3)	< 0.001
BMI after dialysis (kg/m^2^)	24.9 (3.7)	24.8 (4.2)	24.1 (1.6)	24.4 (5.0)	0.934
Albumin (g/dL)	4.1 (0.4)	4.2 (0.4)	4.0 (0.4)	4.0 (0.3)	0.108
C-reactive protein (mg/L)*	2.3 (0.9–6.8)	5.0 (2.6–10.4)	1.9 (0.9–6.6)	1.0 (0.2–1.3)	< 0.001
Predialysis creatinine (mg/dL)	11.1 (2.6)	8.6 (2.4)	7.3 (2.4)	9.1 (2.2)	< 0.001
Cholesterol (mmol/L)	3.7 (1.1)	–	4.7 (1.2)	4.5 (1.1)	0.001
Body Surface Area (m^2^)	1.9 (0.2)	1.8 (0.2)	1.7 (0.2)	1.7 (0.2)	< 0.001

Values are n (%) for categorical variables, and mean (standard deviation) or *median (Q1- Q3) for continuous variables

*CVD* cardiovascular disease, *BP* blood pressure, *AVF* arteriovenous fistula, *BMI* body mass index

## Appendix 2

**Table 5:** Hazard ratio and 95% confidence intervals of transplantation comparing HDF versus. HD stratified by study

		CONTRAST Hazard ratio (95%-CI)	*p*-value for interaction	ESHOL Hazard ratio (95%-CI)	*p*-value for interaction
**Overall**		1.06 (0.77–1.45)	–	1.19 (0.88–1.61)	–
**Sex**	Male	1.00 (0.66–1.52)		1.09 (0.75–1.58)	
	Female	1.14 (0.69–1.86)	0.70	1.43 (0.85–2.40)	0.40
**Age**	< 60 years	1.47 (0.98–2.19)		1.27 (0.85–1.88)	
	≥ 60 years	0.65 (0.38–1.11)	0.02	1.08 (0.68–1.72)	0.61
**Diabetes**	No	1.02 (0.71–1.46)		1.16 (0.83–1.61)	
	Yes	1.42 (0.68–2.99)	0.43	1.29 (0.60–2.78)	0.80
**History of CVD**	No	1.16 (0.81–1.67)		1.29 (0.92–1.80)	
	Yes	0.80 (0.40–1.59)	0.35	0.97 (0.48–1.94)	0.46
**Albumin**	< 4 g/dL	0.98 (0.57–1.69)		1.56 (0.81–3.02)	
	≥ 4 g/dL	1.07 (0.72–1.60)	0.80	1.12 (0.79–1.59)	0.38
**Dialysis vintage**	< 30 months	1.28 (0.83–1.98)		1.07 (0.74–1.55)	
	≥ 30 months	0.85 (0.52–1.38)	0.21	1.47 (0.87–2.47)	0.33
**Fistula**	No	1.65 (0.66–4.08)		1.58 (0.45–5.48)	
	Yes	1.00 (0.71–1.41)	0.32	1.19 (0.87–1.63)	0.67

## Appendix 3

### List of study investigators

The HDF Pooling project group comprises of all principal investigators of the individual studies that participate in the HDF Pooling project.

**ESHOL investigators:** Francisco Maduell, Francesc Moreso, Mercedes Pons, Rosa Ramos, Josep Mora-Macià, Jordi Carreras, Jordi Soler, Ferran Torres, Josep M. Campistol, and Alberto Martinez-Castelao M. Pons, B. Insensé, C. Perez, and T. Feliz (CETIRSA, Barcelona); R. Ramos, M. Barbetta, and C. Soto (Hospital San Antonio Abad, Vilanova i la Geltru); J. Mora, A. Juan, and O. Ibrik (Fresenius Medical Care,Granollers); A. Foraster and J. Carreras (DiaverumBaix Llobregat, Hospitalet); F. Moreso, J. Nin, and A. Fernández (Fresenius Medical Care, Hospitalet); J. Soler, M. Arruche, C. Sánchez, and J. Vidiella (Fresenius Medical Care, Reus); F. Barbosa, M. Chiné, and S. Hurtado (Fresenius Medical Care Diagonal, Barcelona); J. Llibre, A. Ruiz, M. Serra, M. Salvó, and T. Poyuelo (CETIRSA, Terrassa); F. Maduell, M. Carrera, N. Fontseré, M. Arias, and Josep M. Campistol (Hospital Clínic, Barcelona); A. Merín and L. Ribera (Fresenius Medical Care Julio Verne, Barcelona); J.M. Galceran, J. Mòdol, E. Moliner, and A. Ramirez (Fundació Althaia, Manresa); J. Aguilera and M. Alvarez (Hospital Santa Tecla, Tarragona); B. de la Torre and M. Molera (Diaverum Bonanova, Barcelona); J. Casellas and G. Martín (Diaverum IHB, Barcelona); E. Andres and E. Coll (Fundació Puigvert, Barcelona); M. Valles and C. Martínez (Hospital Josep Trueta, Girona); E. Castellote (Hospital General, Vic); J.M. Casals, J. Gabàs, and *M. Romero* (Diaverum, Mataró); A. Martinez-Castelao and X. Fulladosa (Hospital Universitari Bellvitge, Hospitalet); M. Ramirez-Arellano and M. Fulquet (Hospital de Terrassa); A. Pelegrí, M. el Manouari, and N. Ramos (Diaverum Verge de Montserrat, Santa Coloma); J. Bartolomé (Centre Secretari Coloma, Barcelona); R. Sans (Hospital de Figueres); E. Fernández and F. Sarró (Hospital Arnau de Vilanova, Lleida); T. Compte (Hospital Santa Creu, Tortosa); F. Marco and R. Mauri (Diaverum Nephros, Barcelona); and J. Bronsoms (Clínica Girona), J.A. Arnaiz, H. Beleta, and A. Pejenaute (UASP Farmacología Clínica, Hospital Clínic Barcelona), F. Torres, J. Ríos, and J. Lara (Biostatistics Unit, School of Medicine, Universitat Autònoma de Barcelona).

**CONTRAST investigators:** P.J. Blankestijn, M.P.C. Grooteman, M.J.Nubé, P.M. ter Wee, M.L. Bots; M.A. van den Dorpel, Canada: Georges-L. Dumont Regional Hospital, Moncton: M. Dorval; CHUM St. Luc Hospital, Montréal: R. Lévesque. The Netherlands: Academic Medical Center, Amsterdam: M.G. Koopman; Catharina Hospital, Eindhoven: C.J.A.M. Konings; Dialysis Clinic Noord, Beilen: W.P. Haanstra; Dianet Dialysis Centers, Utrecht: M. Kooistra and B. van Jaarsveld; Fransiscus Hospital, Roosendaal: T. Noordzij; Gelderse Vallei Hospital, Ede: G.W. Feith; Groene Hart Hospital, Gouda: H.G. Peltenburg; Haga Hospital, The Hague: M. van Buren; Isala Clinics, Zwolle: J.J.G. Offerman; Jeroen Bosch Hospital, Hertogenbosch: E.K. Hoogeveen; Maasland Hospital, Sittard: F. de Heer; Maasstad Hospital, Rotterdam: P.J. van de Ven; Martini Hospital, Groningen: T.K. Kremer Hovinga; Medical Center Alkmaar: W.A. Bax; Onze Lieve Vrouwe Gasthuis, Amsterdam: J.O. Groeneveld; Oosterschelde Hospital, Goes: A.T.J. Lavrijssen; Rijnland Hospital, Leiderdorp: A.M. Chrander-Van der Meer; Rijnstate Hospital, Arnhem: L.J.M. Reichert; Slingeland Hospital, Doetinchem: J. Huussen; St. Elisabeth Hospital, Tilburg: P.L.Rensma; St. Fransiscus Gasthuis, Rotterdam: Y. Schrama; University Medical Center St. Radboud, Nijmegen: H.W. van Hamersvelt; University Medical Center Utrecht, Utrecht: W.H. Boer; VieCuri Medical Center, Venlo: W.H. van Kuijk; VU University Medical Center, Amsterdam: M.G. Vervloet; Zeeuws-Vlaanderen Hospital, Terneuzen: I.M.P.M.J. Wauters. Norway: Haukeland University Hospital, Bergen: I. Sekse.

**Turkish HDF Study investigators:** Ercan Ok, Gulay Asci, Huseyin Toz, Ebru Sevinc Ok, Fatih Kircelli, Mumtaz Yilmaz, Ender Hur, Meltem Sezis Demirci, Cenk Demirci, Soner Duman, Ali Basci, Siddig Momin Adam, Ismet Onder Isik, Murat Zengin, Gultekin Suleymanlar, Mehmet Emin Yilmaz and Mehmet Ozkahya Pinar Ergin, Alfert Sagdic, Erkan Kayali, Can Boydak, Taskin Colak, Sihli Caliskan, Hakan Kaplan, Hasibe Ulas, Sait Kirbiyik, Hakan Berktas and Necati Dilbaz.

**French HDF Study investigators:** Bernard Canaud, Jean-Paul Cristol, Marion Morena, Hélène Leray-Moragues, Leïla Chenine, Marie-Christine Picot, Audrey Jaussent, Claire Belloc, Mélodie Lagarrigue (University Hospital Center, Montpellier), Lotfi Chalabi (AIDER, Montpellier), Alain Debure, Messaoud Ouziala, Jean-Jacques Lefevre (ATS, St Denis), Damien Thibaudin, Hesham Mohey, Christian Broyet, Aida Afiani, (University Hospital Center, Saint Etienne), Marie-Odile Serveaux, Laure Patrier (University Hospital Center, Nîmes), François Maurice, Jean-Pierre Rivory (CHL, Castelnau le Lez), Philippe Nicoud (La Vallée Blanche center, Chamonix), Claude Durand, Michel Normand (Saint Martin polyclinic, Pessac), Bruno Seigneuric (University Hospital Center, Toulouse), Eric Magnant (Centre Hémodialyse Provence, Aix en Provence), Lynda Azzouz (Artic 42, St Priest en Jarez), Mohamed Shariful Islam, Sandor Vido (University Hospital Center, Nice), Hilaire Nzeyimana, Danièle Simonin (ECHO Michel Ange center, Le Mans), Yamina Azymah, Ibrahim Farah, Jean-Philippe Coindre (Hospital center, Le Mans), Olivier Puyoo, Marie-Hélène Chabannier, Richard Ibos, Fabienne Rouleau (Centre Néphrologique d’Occitanie, Muret), Carlos Vela, Josiane Joule (Hospital center, Perpignan), François Combarnous (Tonkin clinic, Villeurbanne), Cécile Turc-Baron, Francis Ducret, Philippe Pointet, Isabelle Rey (Hospital center, Annecy), Jacky Potier (Hospital center, Cherbourg), Jean- Christophe Bendini, Franck Perrin (St Georges clinic, Nice), Kristian Kunz (University Hospital Center, Strasbourg), Gaëlle Lefrancois, Angélique Colin, Sophie Parahy, Irima Dancea, Stéphanie Coupel, Angelo Testa (ECHO center, Rezé), Philippe Brunet, Gaétan Lebrun, Dominique Jaubert (University Hospital Center AP-HM, Marseille), Catherine Delcroix, Frédéric Lavainne, Anne Lefebvre (University Hospital Center, Nantes), Marie-Paule Guillodo, Dominique Le Grignou (AUB, Brest), Assia Djema (Hospital center, Cholet), Mehadji Maaz, Sylvie Chiron (Hospital center, Colmar), Maxime Hoffmann, Pascale Depraetre (Louvière clinic, Lille), Atman Haddj-Elmrabet, Véronique Joyeux (University Hospital Center, Rennes), Dominique Fleury, Laurence Vrigneaud, Vincent Lemaitre (Hospital center, Valenciennes), Didier Aguilera, Abdallah Guerraoui (Hospital center, Vichy), Alain Cremault, Achour Laradi, Francois Babinet (ECHO Pole Santé Sud center, Le Mans).

The writing executive committee, whose membership did not include representatives of the financial contributors, has final responsibility for the interpretation of the data, the preparation of the manuscript and the decision to submit for publication. The executive committee vouches for the validity and completeness of the reported data.

## Appendix 4

### Funding

The HDF Pooling project was designed, conducted, and analysed independently of the financial contributors of the individual studies as listed below. Study data were collected and retained by the investigators and were not available for the financial contributors of the individual studies. SAEP and the meetings of the representatives of the combined authors of the four studies were financially supported by the EuDial working group. EuDial is an official working group of the European Renal Association – European Dialysis Transplant Association (ERA-EDTA, http://era-edta.org/eudial/European_Dialysis_Working_Group.html. No industry funding was received for any part of or activity related to the present analysis.

The Turkish HDF study was supported by European Nephrology and Dialysis Institute with an unrestricted grant. The study was performed in Fresenius Medical Care haemodialysis clinics in Turkey. ESHOL was supported by The Catalan Society of Nephrology and by grants from Fresenius Medical Care and Gambro through the Catalan Society of Nephrology. The CONTRAST study was supported by a grant from the Dutch Kidney Foundation (Nierstichting Nederland Grant C02.2019), and unrestricted grants from Fresenius Medical Care, Netherlands, and Gambro Lundia AB, Sweden. Additional support was received from the Dr. E.E. Twiss Fund, Roche Netherlands, the International Society of Nephrology/ Baxter Extramural Grant Program, and the Netherlands Organization for Health Research and Development (ZONMw Grant 170,882,802). The French HDF study was supported by a national grant from the Health Ministry (Programme Hospitalier de Recherche Clinique, PHRC) as a mean to improve care and outcome of elderly chronic disease patients.
